# Muscle Belly Gearing Positively Affects the Force–Velocity and Power–Velocity Relationships During Explosive Dynamic Contractions

**DOI:** 10.3389/fphys.2021.683931

**Published:** 2021-08-12

**Authors:** Andrea Monte, Matteo Bertucco, Riccardo Magris, Paola Zamparo

**Affiliations:** Department of Neurosciences, Biomedicine and Movement Sciences, University of Verona, Verona, Italy

**Keywords:** gear ratio, ultrasound, mechanical power, concentric contraction, fascicle velocity, muscle geometry

## Abstract

Changes in muscle shape could play an important role during contraction allowing to circumvent some limits imposed by the fascicle force–velocity (F–V) and power–velocity (P–V) relationships. Indeed, during low-force high-velocity contractions, muscle belly shortening velocity could exceed muscle fascicles shortening velocity, allowing the muscles to operate at higher F–V and P–V potentials (i.e., at a higher fraction of maximal force/power in accordance to the F–V and P–V relationships). By using an ultrafast ultrasound, we investigated the role of muscle shape changes (vastus lateralis) in determining belly gearing (muscle belly velocity/fascicle velocity) and the explosive torque during explosive dynamic contractions (EDC) at angular accelerations ranging from 1000 to 4000°.s^–2^. By means of ultrasound and dynamometric data, the F–V and P–V relationships both for fascicles and for the muscle belly were assessed. During EDC, fascicle velocity, belly velocity, belly gearing, and knee extensors torque data were analysed from 0 to 150 ms after torque onset; the fascicles and belly F–V and P–V potentials were thus calculated for each EDC. Absolute torque decreased as a function of angular acceleration (from 80 to 71 Nm, for EDC at 1000 and 4000°.s^–1^, respectively), whereas fascicle velocity and belly velocity increased with angular acceleration (*P* < 0.001). Belly gearing increased from 1.11 to 1.23 (or EDC at 1000 and 4000°.s^–1^, respectively) and was positively corelated with the changes in muscle thickness and pennation angle (the changes in latter two equally contributing to belly gearing changes). For the same amount of muscle’s mechanical output (force or power), the fascicles operated at higher F–V and P–V potential than the muscle belly (e.g., P–V potential from 0.70 to 0.56 for fascicles and from 0.65 to 0.41 for the muscle belly, respectively). The present results experimentally demonstrate that belly gearing could play an important role during explosive contractions, accommodating the largest part of changes in contraction velocity and allowing the fascicle to operate at higher F–V and P–V potentials.

## Introduction

The ability of the human skeletal muscle to produce high values of force as quickly as possible is important for movements that involve explosive tasks or rapid postural adjustments such as those encountered in sport performance or during balance recovery (Aagaard et al., 2002; Maffiuletti et al., 2016). Despite the important role of explosive dynamic contractions (EDC), the determinants of explosive torque are often investigated during fixed-end contraction (Aagaard et al., 2002; Maffiuletti et al., 2016; Massey et al., 2017; Del Vecchio et al., 2018, 2019; Dideriksen et al., 2020; Monte, 2020; Monte and Zignoli, 2020). Indeed, only two studies tried to understand the underpinning mechanisms of the torque rise in EDC so far. Tillin et al. (2018) investigated the effects of contraction speed on torque production during the rising phase of the torque–time curve (from start to 150 ms after torque onset). These authors provided evidence that the normalised EMG activity and neural efficacy during explosive concentric contraction were similar among contraction speeds, suggesting that the effect of speed on normalised explosive torque is not dependent on neuromuscular activation but could be an intrinsic property of contracting myofibers. However, fascicle behaviour was not investigated, and the authors suggested that further research would be needed to assess whether structural and mechanical factors could also contribute to the observed differences in explosive torque production (Tillin et al., 2018). Hahn et al. (2017) investigated torque production in order to provide insight into the influence of angular acceleration on maximal torque at matched angular position and velocity. They showed that the absolute torque generated during dynamic explosive contractions (from 10 to 4000°/s^2^) was lower than that obtained during fixed-end contractions but, in accordance with Tillin et al. (2018), that the normalised torque generated during dynamic explosive contractions was larger than in fixed-end conditions (and increased with increasing angular acceleration). Hahn et al. (2017) argued that one possible source of difference between the two types of contractions could be attributed to the behaviour of the elastic components or to the decoupling behaviour between muscle belly and muscle fascicles.

In pennate muscles, dynamic shape changes are thought to play an important functional role in modulating the mechanical performance of skeletal muscle by enhancing the range of velocities over which a fascicle can shorten (Azizi et al., 2008). During concentric contractions, fascicles shorten and rotate to greater pennation angles and this contractile behaviour could be accompanied by an increase in muscle thickness, decoupling the length changes of the fascicles from the length changes of the muscle belly in a process known as belly gearing (Azizi et al., 2008; Randhawa et al., 2013; Holt and Azizi, 2016; Holt et al., 2016). Studies have shown that gearing changes in response to the mechanical demands of the task, thereby allowing fascicles to operate at velocities more favourable for power production across a range of MTU velocities (e.g., at higher F–V and P–V potentials: at higher fractions of maximal torque/power in accordance to the F–V and P–V relationships) (Wakeling et al., 2011; Eng et al., 2018; Roberts et al., 2019). For example, low-force high-velocity contractions are associated with larger fascicle rotations and high gearing, whereas high-force low-velocity contractions are associated with smaller fascicle rotations and low gearing (Azizi et al., 2008; Dick and Wakeling, 2017, 2018). Such a phenomenon suggests that belly gearing is higher during rapid movements and could allow to circumvent the limits imposed by the fascicle’s F–V, increasing the force/torque rise. Indeed, the force generated is lower the higher the velocity of movement, in agreement with the F–V relationships. However, to date, our understanding of dynamic muscle shape changes during explosive dynamic contraction is limited and their role in determining the torque rise during explosive concentric contraction is actually still understood.

Hence, in this study, we combined ultrafast ultrasound measurements, dynamometric, and EMG measurements to experimentally investigate the (possible) effect of belly gearing on the F–V and the P–V relationships during explosive concentric contractions. Finally, we aimed to investigate the determinants of belly gearing by analysing the muscle shape changes.

Since changes in muscle thickness are inversely related to the level of contractile force (Eng et al., 2018), we expected muscle thickness changes during contraction to increase as a function of contraction velocity (according to the F–V relationship), allowing fascicle rotation and belly gearing to increase. The increase in belly gearing should allow the muscle fascicles to operate under a more favourable portion of their F–V and P–V relationships in comparison to the muscle belly (Wakeling et al., 2011), increasing the contraction velocity without a significant loss in force production. Therefore, we hypothesised the F–V and P–V potentials of the muscle belly to be lower than those of the fascicles.

## Materials and Methods

### Participants

Twenty-two moderately active healthy men (recreational runners training once/twice per week; age: 25 ± 3 years; body mass: 76 ± 3.5 kg; stature: 1.76 ± 0.03 m) participated in this study. The participants did not report any type of neuromuscular injury during the 6 months before the experiments. All participants received written and oral instructions before the study and gave their written informed consent to the experimental procedure. The experimental protocol was approved by the Ethical Committee of the University of Verona (protocol number: 2019-UNVRCLE-0193291) and was performed in accordance with the Helsinki Declaration.

### Experimental Design

Each subject participated in three sessions. In the first visit (preliminary session), a series of sub-maximal voluntary concentric contractions were performed for familiarisation.

In the first experimental session, the torque–angle and torque–angular velocity relationships of the knee extensors were experimentally determined by means of maximum fixed-end contraction (MVC) and iso-velocity tests. The values of torque were then converted into force values by knowing the patellar tendon moment arm. Finally, using an ultrasound apparatus we determined the length changes of the fascicles and muscle belly of vastus lateralis (VL), in order to obtain their corresponding force–length, force–velocity, and power–velocity relationships. After 20 min of passive rest, explosive contractions were recorded in dynamic concentric conditions (EDC) at different, and constant, accelerations of 1000, 2000, 3000, and 4000°.s^–2^ (in randomised order).

In the second experimental session, the procedures of the first session were repeated (with the exception of the assessment of the torque–angle and torque–angular velocity relationships) to check the reliability and the sensitivity of the ultrasound measurements.

During both experimental sessions, the architectural changes of the VL were analysed. The knee joint torque and the EMG activity of VL and biceps femoris (BF) were also recorded.

#### Explosive Contraction Set-Up

To properly compare explosive torque production in dynamic conditions the muscle’s contractile capability should be the same/similar. To do that, each subject was positioned in order to express the same F–L potential (i.e., fraction of the maximum force in accordance with the F–L curve of fascicles) during each explosive-dynamic contraction (see Table 1). The F–L potential expresses the operating length of the muscle fascicles with respect to the F–L curve and can be calculated based on the fascicle operating range throughout the F–L curve during contraction (see Nikolaidou et al., 2017; Bohm et al., 2019; Monte et al., 2020).

**TABLE 1:** Kinematic data obtained during the first 150 ms after torque onset.

**Variable**	**EDC_1000_**	**EDC_2000_**	**EDC_3000_**	**EDC_4000_**
Peak angular velocity after 150 ms (°.s^–1^)^§^	147 ± 4^+^^∧#^	298 ± 3°^∧⁢#^	446 ± 6^+^°^#^	597 ± 7^+∧^°
Angular velocity after 75 ms (°.s^–1^)^§^	73 ± 3^+^^∧#^	149 ± 3°^∧#^	223 ± 5^+^°^#^	298 ± 6^+∧^°
Knee angle at voluntary torque onset (°)^§^	70 ± 3^+∧#^	76 ± 4°^∧#^	81 ± 3^+^°^#^	87 ± 3^+∧^°
Knee angle at 75 ms from torque onset (°)^§^	64 ± 2	65 ± 3	64 ± 3	65 ± 3
Knee angle ROM (°)^§^	11 ± 3+∧#	23 ± 5°^∧#^	34 ± 5^+^°^#^	45 ± 4^+∧^°

*Values are mean ± SD. The angular velocity at 75 ms is approximately half of the maximum angular velocity reached at 150 ms, confirming the condition of constant angular acceleration. For all these parameters, statistical results refer to one-way repeated measure ANOVA. ROM: range of motion; absolute explosive torque: average values of torque obtained in the first 150 ms after torque onset; normalised EMG: average EMG values in the first 150 ms after torque onset, expressed as a percentage of the EMG value at maximum voluntary EMG amplitude.*

*^§^Significant main effect of acceleration.*

*^°^Significant difference compared with EDC_1000_.*

*^+^Significant difference compared with EDC_2000_.*

*^∧^Significant difference compared with EDC_3000_.*

*^#^Significant difference compared with EDC_4000_.*

**FIGURE 1:**
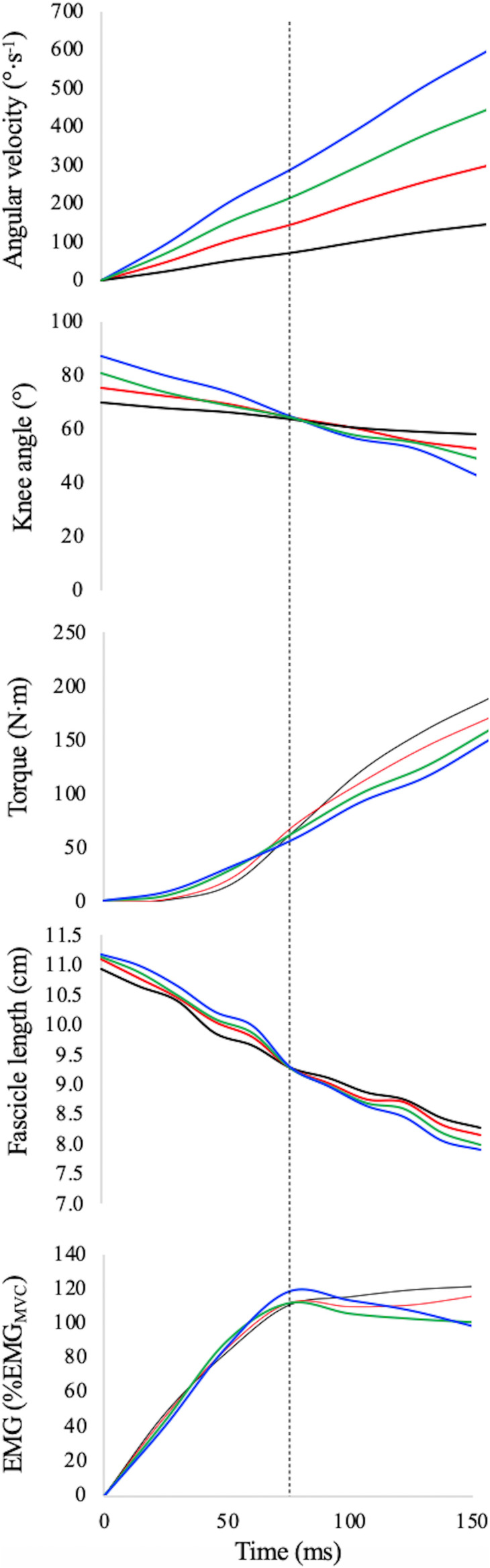
Knee angular velocity, knee angle, absolute explosive torque, absolute fascicle length, and normalised EMG activity of vastus lateralis during explosive contractions (black line: EDC_1000_, red line: EDC_2000_, green line: EDC_3000_, and blue line: EDC_4000_) for a typical subject. Target angle as well as optimal fascicle length (for this subject: 65° and 9.3 cm, respectively) was reached 75 ms after torque onset at all contraction velocities (dotted vertical line).

Explosive contractions are typically investigated in the first 150 ms after contraction onset, due to the importance of this time window in athletic events (e.g., ground contact in sprint running) and daily life activities (balance recovery). For each participant, the range of motion (ROM) during EDC contractions was set as to present the optimal fascicle length “in the middle” of the 150 ms time window (Table 1): these contractions were, thus, set to start 75 ms before reaching the target length (i.e., the optimal length obtained from the F–L relationship using the fixed-end contractions at different joint angles), independently from the values of angular acceleration (see Figure 1). Therefore, each participant had his own dynamometric configuration. In order to reach this goal two issues were taken into account: (i) with increasing angular acceleration the time necessary to reach the target length decreases; therefore the ROM had to be determined experimentally during pilot testing and each EDC contraction started from a different knee angle (see Table 1); (ii) to avoid that the dynamometer stops accelerating as soon as the set velocity is reached, the dynamometer peak angular velocity was set to 660°.s^–1^ (see Hahn et al., 2017 for further details).

Several pilot tests were performed to establish and check the experimental procedures for the explosive contractions. Even though ROM and torque onset were different among contractions, the target length of each participants (optimal length, that corresponds at an optimal angle of ∼65°) during EDC contractions was reached 75 ms after torque onset in all acceleration conditions (see Table 1). The primary aim of this standardisation was to allow the muscles to work within a similar force–length potential in all contractions (see Table 1). The differences in fascicle length during the first 150 ms of contraction are, thus, not expected to have a significant effect on mechanical output; the observed differences could be essentially attributed to the differences in contraction velocity imposed by the different angular acceleration.

### Data Collection

#### F–L, F–V, and P–V Relationships

The procedures to determine the force–length, force–velocity, and power–velocity relationships are similar to those reported in previous studies (Tillin et al., 2012b, 2018; Nikolaidou et al., 2017; Monte et al., 2020), which involved: (1) the assessment of the torque–angle and torque–angular velocity by means of the dynamometer; (2) the conversion of torque into force values using patellar tendon moment arm; and (3) the combination of these force values with ultrasound data, to obtain the force–length, force–velocity, and power–velocity relationships of the fascicles and of the muscle belly.

Briefly, the participants were secured on a dynamometer (Biodex System 4 Pro, United States) by means of a trunk and pelvic strap, the arms crossed in front of the chest. Due to the knee joint rotation that occurs during knee extension contractions from rest to MVC (Tsaopoulos et al., 2006), the knee and the dynamometer axis of rotation were aligned at 65° of knee flexion during MVC (Bakenecker et al., 2019). Therefore, the alignment was performed at higher torque values than during the dynamic explosive contractions. This implies that the soft tissues displacement was different in the two cases (maximum vs. explosive contraction). However, during pilot tests, we checked for different configurations (i.e., different alignment during sub-maximal contraction) and no significant differences in offset were observed (i.e., differences in the alignment between centers of rotation could not affected our results). Furthermore, to avoid possible inaccuracies in knee angle measurements due to soft tissue deformation we used 2D video analysis to calculate the actual knee joint angle during all contractions on the basis of the following markers: iliac spine, greater trochanter of the opposite side, lower portion of the patella (patellar tendon origin), upper anterior surface of the tibia (patellar tendon insertion), and knee centre of rotation. The marker positions were recorded by means of a Casio Exilim Camera (200 fps, Casio Computer Co., Ltd., Tokyo, Japan) and analysed with a video processing software (Tracker v4.0). The camera was positioned on the left side of the subject, perpendicular to the thigh longitudinal axis. To eliminate any radial distortion, a rectilinear filter was applied during marker tracking on the video frames. The resultant marker trajectories were smoothed using a forward and reverse second order low pass Butterworth filter (cut-off: 15 Hz). Based on these kinematic data, the patellar tendon moment arm could be calculated [as proposed by Bakenecker et al. (2019)].

After a warm-up based on sub-maximal fixed-end and iso-velocity contractions, the torque–angle relationship was obtained based on eight maximum fixed-end contractions (MVCs) at different joint angles (from 90° to 20° where 0° = knee fully extended) with 10° intervals, while the torque–angular velocity relationship was obtained based on values of maximum torque recorded during the iso-velocity phase at the angular velocities of 60, 90, 180, 280, and 360°.s^–1^.

During the MVCs and the iso-velocity tests, the subjects were instructed to push “as hard as possible” for 4–5 s (for the MVCs) or over the entire ROM (for the iso-velocity trials). Two maximal fixed-end contractions were performed for each joint angle, with 2 min of recovery in between, whereas for the iso-velocity trials three consecutive contractions (with 30 s of recovery in between) were performed at each velocity with 2 min of recovery between velocities. For each iso-velocity trial, participants were instructed to extend their knee as hard as possible from ∼0.5 s before the start of the set to the end of the set. This protocol pre-loaded the muscles facilitating maximal voluntary neuromuscular activation, and therefore maximum torque, throughout the entire ROM (Tillin et al., 2012a, 2018).

During all contractions, fascicle length changes were recorded by means of an ultrafast ultrasound apparatus with a 5.5 cm linear array probe operating at 1000 fps (Supersonic, Aixplorer, France). The probe was fixed to the skin by means of a plastic strap approximately at 50% of the femoral length, aligned on the muscle belly and corrected with respect to the superficial and deep aponeurosis, in order to have a clear image of the perimysial connective intramuscular tissue, that it is indicative of the muscle fascicle structure. The probe was never removed during the entire experimental session and a marker on the skin was drawn to reposition the probe in the same position during the second experimental session (48 h later).

Finally, the EMG activity of the VL and BF were recorded during each contraction by means of two bipolar Ag-AgCl electrodes. The electrodes were attached over the muscle belly according to the SENIAM recommendations (Hermens et al., 2000). The raw EMG data were recorded at 1000 Hz with a PowerLab System (PowerLab, ADInstruments, Dunedin, New Zealand).

All devices were synchronised with an external manual trigger (5 V) and all data were collected at 1000 Hz.

#### Explosive Contractions

During the explosive dynamic concentric contractions the participants were instructed to extend their knee “as fast and as hard” as possible with an emphasis on “fast” (Sahaly et al., 2001) in four separate conditions: 1000 (EDC_1000_), 2000 (EDC_2000_), 3000 (EDC_3000_), and 4000°.s^–2^ (EDC_4000_). For each condition, the lever arm angular acceleration was preprogrammed using a real-time control custom program (LabView v.10) to increase the Biodex capability, as proposed by Van Roie et al. (2020). At least 15 contractions were recorded for each condition; 1 min of recovery was imposed after each contraction and their order was randomised.

As previously suggested by Tillin et al. (2018), in concentric conditions the dynamometric arm moved slowly (10°.s^–1^) from the maximum knee joint flexion angle to the angular position at which the acceleration phase starts (which was different for each acceleration condition, see knee angle at voluntary torque onset in Table 1). In all concentric contractions, the starting angle for each participant was selected in order to have 75 ms of contraction before and after the target joint angle: the angle at which his optimal fascicle length was observed (about 65°, see knee angle at 75 ms from torque onset in Table 1 and Figure 1).

During each dynamic explosive concentric contraction, participants were instructed to avoid any countermovement (negative/flexor torque). For all concentric conditions, the real-time knee angle signal (Biodex output) was displayed on a computer monitor in front of the participants. In this way, the subject could control the start of the acceleration phase (Tillin et al., 2018).

For each condition, 15 “good” contractions were selected. The quality of the contraction was controlled in real-time by an operator using the LabChart software, based on the EMG and the torque signals. Exclusion criteria were similar to those reported by Tillin et al. (2018) and Tillin et al. (2012a). Therefore, the EDC contractions were excluded if: (1) baseline torque exceeded ± 2 Nm in the 2 s preceding active-torque onset; (2) baseline torque in the 100 ms preceding active torque changed by more than 2.5 Nm; (3) torque onset occurred earlier than 20 ms (all conditions) or later than 30 ms into the acceleration phase. Torque onset in all conditions was defined as the point at which the first derivative of the active torque–time curve crossed zero for the last time (Tillin et al., 2012a). During these procedures, the total torque generated by the knee extensors was corrected for the gravitational torque effects (determined during a passive joint rotation driven by the dynamometer).

As in the case of the MVCs and iso-velocity tests, ultrasound data, EMG activity, and kinematic data were recorded at 1000 Hz. The location of the electrodes, as well as the location of the ultrasound probe, was the same used for the MVCs and iso-velocity trials.

### Data Analysis

#### F–L, F–V, and P–V Relationships

The total torque generated by the knee extensors was corrected for the gravitational torque effects (determined during a passive joint rotation driven by the dynamometer) and for the EMG–torque relationships of the antagonist muscles (BF) (see Kellis and Baltzopoulos, 1996, 1998). The dynamometer torque output was also corrected for inertia and passive torque. The moment of inertia (i.e., shank, foot, and dynamometric arm) was estimated as proposed by Farcy et al. (2014) and Hauraix et al. (2017) and the passive torque was calculated and subtracted to the measured values as proposed by Tillin et al. (2012a, b). Finally, the torque generated by the knee extensors was converted into force by knowing the patellar tendon moment arm [as proposed by Bakenecker et al. (2019)].

For the ultrasound measurements, a validated semi-automatic tracking algorithm was used to quantify the muscle fascicle length and pennation angle frame by frame (Gillett et al., 2013). The length of muscle fascicles was defined as the distance along the fascicles between the deep and superficial aponeuroses, whereas the pennation angle (α) was defined as the angle between the collagenous tissue and the deep aponeurosis (Franchi et al., 2018; Monte and Zamparo, 2019). Since VL fascicles could be longer than the field of view of the ultrasound probe, the automatic software performed a linear extrapolation of the fascicle length, as reported by others (Randhawa et al., 2013; Hahn et al., 2017; Nikolaidou et al., 2017; Monte et al., 2020). As discussed by Franchi et al. (2020), this extrapolation method may be suitable for estimating fascicle length in muscles that present a relatively linear fascicle arrangement, or a consistent fascicle curvature, as in the case of the middle region of VL. Furthermore, at the end of the auto-tracking, every frame of the tracked fascicle lengths and pennation angles was visually examined to check the algorithm accuracy. Whenever the fascicle length or pennation angle was deemed inaccurate, the two points defining the muscle fascicles were manually repositioned.

The EMG signal was always analysed starting from force onset; in the fixed-end and iso-velocity contractions the EMG signal was smoothed to match the root mean square (RMS) epochs used for the explosive voluntary contractions (Tillin et al., 2012a, 2018). The EMG data at maximum voluntary force (MVF) was then calculated from the average RMS amplitude over a 10 ms epoch either at MVF for each fixed-end knee angle, or at any given knee angle for the best MVC at each velocity (Tillin et al., 2012a,b, 2018).

**FIGURE 2:**
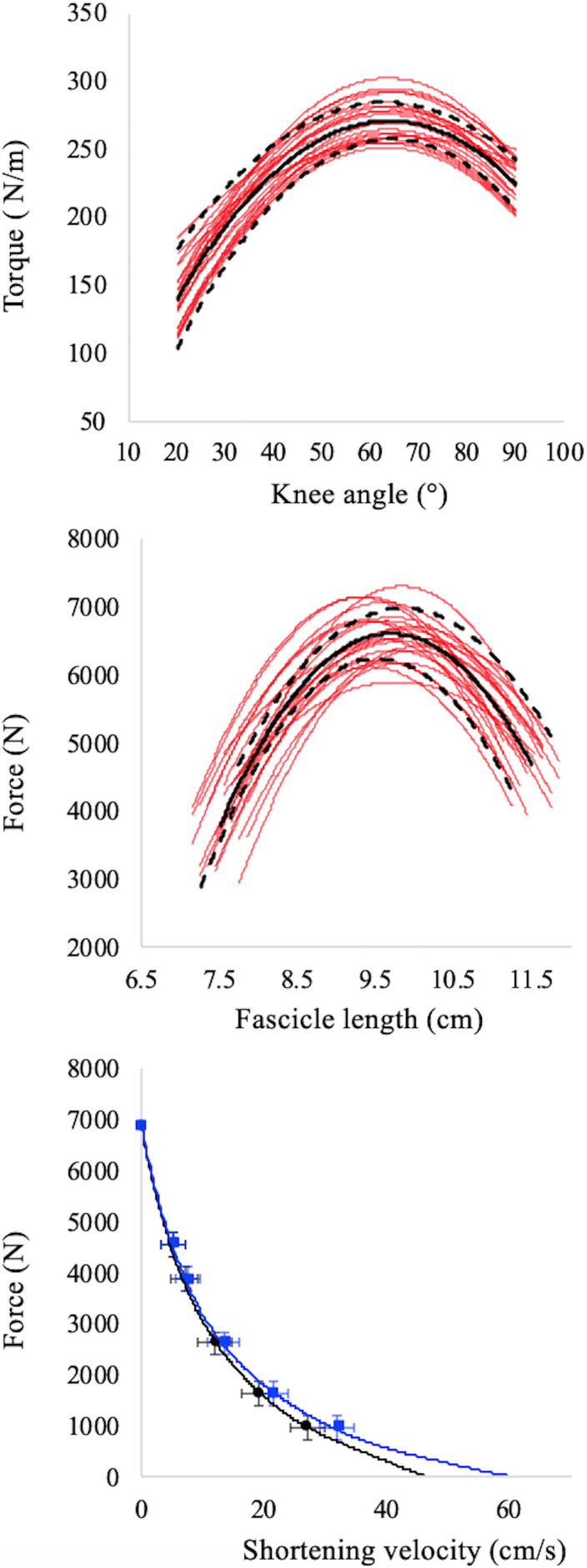
Torque–angle (upper panel), force–fascicle length (middle panel), force–fascicle velocity (black symbols, lower panel), and force–muscle belly velocity (red symbols, lower panel) relationships. Red lines in upper and middle panels refer to individual data while the black continuous and dashed lines refer to average values and standard deviation, respectively. The apex of the F–L relationship (middle panel) is the maximum isometric torque in correspondence of which the optimal length is calculated. Data points in the F–V relationships (lower panel) represent mean ± SD for all subjects. The intercepts on the *X* axes of the F–V relationships (lower panel) represent maximum fascicle (black curve) and belly (red curve) shortening velocity. Note that force values refer the whole muscle but velocities are measured independently in the muscle belly and fascicles.

To reconstruct the force–angle relationship, the highest value of force recorded at each knee angle was taken as the MVF for that angle (see Figure 2, upper panel). Furthermore, the fascicle length obtained at MVF was measured and used to reconstruct the F–L relationships (see Figure 2, middle panel). Therefore, based on MVF data and muscle fascicle length collected at each joint angle, the F–L relationship was also determined for each subject (Monte et al., 2020). The maximum isometric voluntary force (F_*max*_) was calculated as the peak of the F–L relationships, while the optimal fascicle length (L_0_) as the VL fascicle length at which F_*max*_ occurs (see Figure 2, middle panel).

To reconstruct the fascicles and muscle F–V relationships the ultrasound data collected during the iso-velocity trials were used. For these procedures, fascicle velocity was obtained as the first derivative of the fascicle length changes, whereas the belly velocity as the first derivative of the belly length changes (obtained as: Fascicle length. cos α, where α is the pennation angle). As calculated, belly length does not represent the entire length of the VL muscle belly but only the projection of the VL fascicle length to the plane of the MTU; this method to estimate muscle belly velocity (Vb), is utilised when the myotendinous junctions is not tracked with a second ultrasound probe, as in our case (Wakeling et al., 2011; Randhawa et al., 2013; Hauraix et al., 2015; Bohm et al., 2019). Therefore, the F–V relationship was determined twice, based on the force–fascicle velocity and force–belly velocity values during the iso-velocity test (see Figure 2, bottom panel). Note that, as proposed by Holt et al. (2016), force values refer to the force of the whole muscles only, whereas muscle and fascicles velocities are measured independently.

These values were fitted using the following exponential equation, which was identified to be most applicable to F–V data measured *in vivo* compared to the classical Hill’s equation (Thomas et al., 1987):

(1) $$v=\left(e^{-\frac{F}{b}}-e^{-\frac{F_{m\,a\,x}}{b}}\right)\,a$$

where *v* is the fascicle/belly velocity (m.s^–1^), *F* is the force value, F_*max*_ the maximal isometric force recorded during the MVC and extrapolated by the polynomial fit and *a* and *b* are experimentally determined constants. Constants *a* and *b* were obtained from the intercept (*a*/*b*) and slope (1/*b*) of the linearised Hill’s plot of (*P*_0_ − *P*)/*V* vs. *P*, where *V* is the angular velocity of shortening (Ferri et al., 2003; Monte et al., 2020). Finally, the intercept value on the abscissa was taken as the maximal fascicle shortening velocity (Vf_*max*_) or maximum belly shortening velocity (Vb_*max*_).

The P–V relationship was determined based on the product of force and muscle belly velocity at any given time point.

#### Explosive Contractions

As in the case of the MVCs and iso-velocity contraction, the joint torque generated by the knee extensors was calculated by taking into account gravitational torque, inertia, passive torque, and the antagonist contribution. Of the 15 recorded contractions, 5 were selected and further analysed: those in which the angle after 75 ms from onset was closer to target (see Figure 1 and Table 1).

Torque onset was defined as the point at which the first derivative of the active torque–time curve crossed zero for the last time (Tillin et al., 2012a, 2018). All the analysed variables (ultrasound, torque, and EMG parameters) were thus averaged over the five analysed contractions in each condition. The off-line analysis was conducted using custom-developed programs in LabVIEW (LabVIEW, v.10, AD Instruments, Sydney, Australia).

The absolute values of explosive torque reported in this study are the average of the torque values in the first 150 ms of explosive contraction (during the rising torque–time curve).

The five selected explosive contractions in each condition were also analysed for EMG. The EMG signal was filtered with a band-pass fourth order Butterworth filter at 20–450 Hz, whereas the onset of muscle activity was detected using a visual, systematic inspection method as suggested by Tillin et al. (2010, 2012b). During each condition, the RMS EMG amplitude of VL was measured with a time window of 10 ms, from EMG onset up to 150 ms. The EMG signal, as determined in EDC contractions as a function of time, was normalised for the maximum EMG amplitude recorded during the F–L and F–V evaluations. Normalised EMG reported in this study are the average of the EMG values in the first 150 ms of explosive contraction (during the rising torque–time curve).

As in the case of the MVCs and iso-velocity contractions, fascicle length and pennation angle for each explosive contraction were recorded with an automatic software (see F–L and F–V analysis); examples of ultrasound images are reported in Supplementary Figure 1. A reliability and sensitivity study of the ultrafast-ultrasound-based measurement of VL fascicle behaviour during explosive contraction revealed very good reliability between single trials, with coefficient of multiple correlations (CMC) ranging from 0.92 to 0.97. Moreover, the average root mean square difference (RMSD) values were about 1.3–0.7 mm for VL fascicle lengths (see Supplementary Tables 1, 2).

Based on the ultrasound data, fascicle velocity (V_*fascicle*_) was calculated as the first derivative of the fascicle length–time curve, whereas the muscle belly velocity (V_*belly*_) was calculated as the first derivative of the belly length (obtained as: Fascicle length ⋅ cos α, where α is the pennation angle). To take into account the possible mismatch among fascicle and muscle belly, the belly gearing (G_*b*_) was calculated as the ratio between belly velocity and fascicle velocity (Wakeling et al., 2011).

The F–V and the P–V potentials (i.e., fraction of the maximum force or power that a muscle could reach during a given shortening velocity) of fascicle and muscle belly were calculated for each dynamic contraction using the F–V and P–V relationships, as well as the instantaneous value of fascicles and belly shortening velocity (Bohm et al., 2019; Monte et al., 2020).

### Statistics

Data normality and homoscedasticity were assessed using Shapiro–Wilk and Leven tests, respectively. A one-way repeated measures ANOVA with acceleration as independent variable (with Tukey’s HSD as *post hoc*) was used to identify significant differences between accelerations for all kinematic variables. Since angular acceleration, and therefore angular velocity, differed between EDCs, a one-way repeated measures ANCOVA with acceleration as independent variable and angular velocity as covariate was used to identify significant differences between accelerations for: fascicle F–L potential, absolute explosive torque, normalised EMG, changes in muscle thickness, changes in pennation angle and belly gearing. Conversely, a two-way repeated measures ANCOVA was used to check the effects of acceleration (four levels: EDC_1000_, EDC2_000_, EDC_3000_, and EDC_4000_) and muscle structure (two levels: fascicle and muscle belly) on: contraction velocity, F–V potentials, and P–V potentials. Angular velocity was used also in this case as covariate. Tukey’s HSD test was used for the *post hoc* comparisons.

Correlations between variables were calculated according to Pearson correlation coefficient. The *R*^2^ values were used to show the percentage of the variance in explosive torque explained by the muscle behaviour. Finally the impact of muscle fascicle behaviour in determining belly gearing as a function of contraction condition was tested by means of the Hotelling’s statistics (Dunn and Clark, 1971). These statistical analyses were performed with SPSS (version 23, IBM Corporation, United States) and R (version 31, R Foundation for Statistical Computing).

## Results

The absolute values of maximum isometric voluntary force (F_*max*_) and optimal fascicle length (L_0_) were 6900 ± 575 N and 9.72 ± 1.24 cm, respectively; while the maximum fascicle and belly shortening velocity were: Vf_*max*_ = 50.9 ± 6.0 cm.s^–1^ and Vb_*max*_ = 64.5 ± 9.5 cm.s^–1^, respectively. The individual data are reported in Supplementary Table 3 along with the knee angle data that correspond to Lo.

### Kinematic, Kinetic, and EMG Data

In our experimental set-up, the starting angle, as well as the total joint displacement (ROM), must differ among contraction conditions (see Figure 1). As shown in Table 1, knee angle at (voluntary) torque onset increased as a function of angular acceleration (main effect ANOVA: *P* < 0.001), as well as ROM and peak velocity after 150 ms. The target angle (corresponding to the optimal fascicle length) was of about 65° in all subjects and conditions.

A significant main effect of angular acceleration (main effect ANOVA: *P* < 0.001) was observed for all the angular variables reported in Table 1, except for the knee angle at 75 ms from torque onset.

The instantaneous values of absolute torque as a function of time are reported in Figure 1 whereas their average values (obtained during the first 150 ms for each condition) are reported in Table 2. Average absolute explosive torque in explosive dynamic contractions were affected by acceleration (main effect one-way ANCOVA: *P* = 0.018): in accordance with the F–V curve, the larger the angular velocity the lower the absolute torque (torque was 80 ± 5 and 71 ± 4 Nm during EDC_1000_ and EDC_4000_, respectively). *Post hoc* tests are reported in Table 2.

**TABLE 2:** Torque, ultrasound, and EMG data obtained during the first 150 ms after force onset. Values are mean ± SD.

**Variable**	**EDC_1000_**	**EDC_2000_**	**EDC_3000_**	**EDC_4000_**
Fascicle F–L potential^§^	0.89 ± 3	0.88 ± 4	0.88 ± 5	0.87 ± 4
Absolute explosive torque (N.m)^§^	80 ± 5^∧#^	76 ± 4°^#^	72 ± 4°	71 ± 4°^+^
Normalised EMG (%EMG_*MV*__*F*_)^§^	84.2 ± 7.2	82.6 ± 8.3	83.9 ± 8.2	82.9 ± 9.1
ΔThickness (cm)^§^	0.10 ± 0.02^∧+#^	0.15 ± 0.03°^∧#^	0.19 ± 0.03°^+#^	0.22 ± 0.03°^+∧^
ΔPennation (౦)^§^	3.94 ± 0.35^∧+#^	4.63 ± 0.42°^∧#^	5.89 ± 0.45°^+#^	7.05 ± 0.51°^+∧^
Vfas (cm.s^–1^)*^§^	22.0 ± 3.1^∧#^	22.9 ± 2.3^#^	23.6 ± 2.9°	25.6 ± 3.1°^+^
Vbelly (cm.s^–1^)*^§^	24.2 ± 3.4^∧+#^	26.1 ± 3.1 °^∧#^	28.1 ± 3.5°^+#^	31.7 ± 3.7 °^+∧^
Belly gearing^§^	1.11 ± 0.09^∧+#^	1.14 ± 0.06°^∧#^	1.18 ± 0.08°^+#^	1.23 ± 0.07°^+∧^
Fascicle F–V potential*^§^	0.12 ± 0.03^∧#^	0.09 ± 0.03^#^	0.09 ± 0.01°	0.07 ± 0.01°^+^
Muscle F–V potential*^§^	0.10 ± 0.03^∧#^	0.08 ± 0.02^#^	0.08 ± 0.01°	0.05 ± 0.01°^+^
Fascicle P–V potential*^§^	0.70 ± 0.05^∧+#^	0.65 ± 0.06°^∧#^	0.62 ± 0.07°^+#^	0.56 ± 0.06°^+∧^
Muscle P–V potential*^§^	0.65 ± 0.06^∧+#^	0.60 ± 0.05°^∧#^	0.52 ± 0.06°^+#^	0.41 ± 0.07 °^+∧^

*For fascicle F–L potential, absolute explosive torque, ΔThickness, ΔPennation, and belly gearing statistical results refer to one-way repeated measure ANCOVA. For V_*fas*_, V_*belly*_, and all F–V and P–V potentials, statistical results refer to two-way repeated measure ANCOVA.*

*Absolute explosive torque: average values of torque obtained in the first 150 ms after torque onset; normalised EMG: average EMG values in the first 150 ms after torque onset, expressed as a percentage of the EMG value at maximum voluntary EMG amplitude; Δ: maximum changes during the 150 ms of contraction. F–L and F–V potential: fraction of the maximal torque that the fascicle or muscle could reached according to their F–L and F–V relationships, respectively. P–V potential: fraction of the maximal torque that the fascicle or muscle could reached according to their P–V relationship.*

*^§^Significant main effect of acceleration. ^∗^Significant main effect of muscle structure (fascicles vs. muscle belly).*

*°Significant difference compared with EDC_1000_.*

*^+^Significant difference compared with EDC_2000_.*

*^∧^Significant difference compared with EDC_3000_.*

*^#^Significant difference compared with EDC_4000_.*

The values of normalised EMG as a function of time are reported in Figure 1. The normalised EMG data were of about 83% of the maximum EMG activity recorded during MVCs and indicate no significant effect of angular acceleration (main effect one-way ANCOVA: *P* = 0.371) (see Table 2).

### Muscle Shape Changes

Data of muscle thickness, pennation angle, contraction velocity, and belly gearing are reported in Table 2. Significant differences (increases) as a function of angular acceleration were observed in muscle thickness, pennation angle, and belly gearing (main effect one-way ANCOVA: *P* < 0.001 in all cases). Changes in muscle thickness increased from 0.1 ± 0.02 to 0.19 ± 0.03 cm, whereas the changes in pennation angle from 3.94 ± 0.35° to 7.05 ± 0.51° during EDC_1000_ and EDC_4000_, respectively.

Two-way repeated measure ANCOVA revealed significant main effect of acceleration (*P* < 0.001) and muscle structures (fascicle vs. muscle belly; *P* < 0.001) in terms of contraction velocity. In particular, muscle belly and fascicle contraction velocity increased as a function of angular acceleration and were significantly different (i.e., muscle belly shortening velocity was always higher compared to fascicle shortening velocity, see Table 2). Belly velocity increased more than the fascicle velocity: +18 vs. +40% from EDC_1000_ and EDC_4000_ for fascicle and belly velocity, respectively. *Post hoc* tests are reported in Table 2.

**FIGURE 3:**
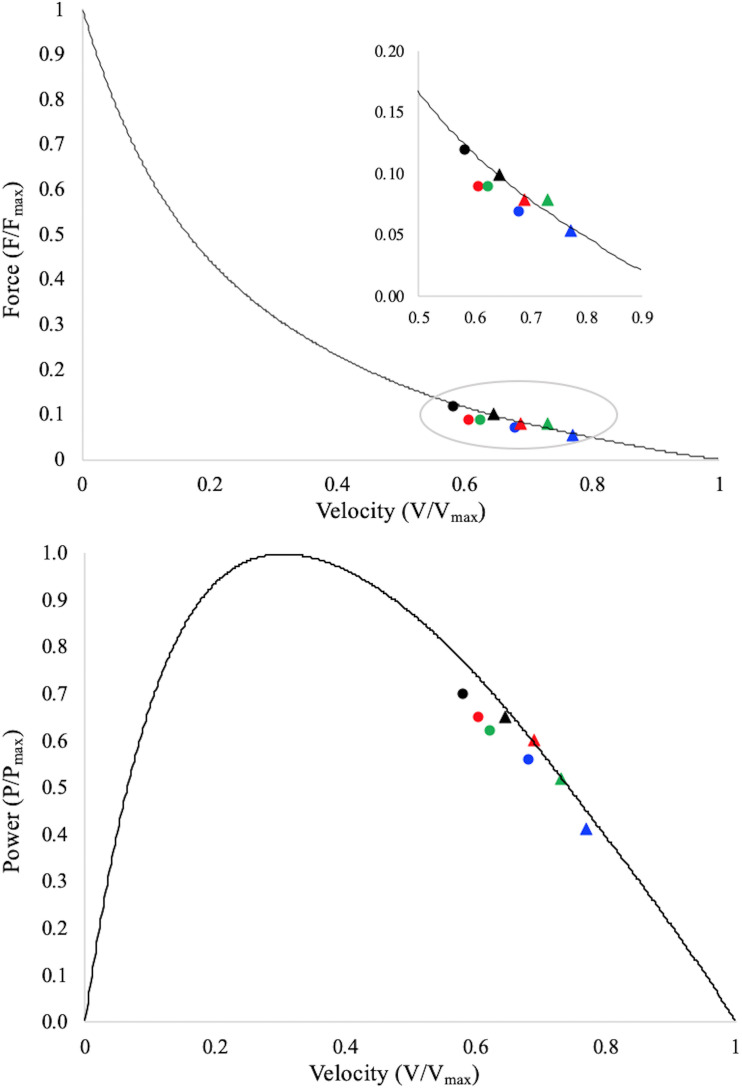
Data points represent average values of fascicle (dots) and muscle belly (triangles) F–V and P–V potentials during the contraction period onto the averaged normalised torque–velocity and power–velocity curves. Torque is normalised to the maximum muscle torque as determined during the maximal isometric contractions; shortening velocity is normalised to the experimentally calculated maximum muscle belly shortening velocity (v_*max*_); power is normalised to the maximum muscle belly mechanical power (P_*max*_). Fascicle potentials are therefore reported as a function of the muscle’s F–V and P–V curves, which allow for a direct comparison between fascicles and muscle belly. Black dot/triangle: EDC_1000_, red dot/triangle: EDC_2000_, green dot/triangle: EDC_3000_, and blue dot/triangle: EDC_4000_. Standard deviations and statistical results are not reported, but could be appreciated in Table 2.

The F–V and the P–V potentials for both muscle fascicles and muscle belly are reported in Figure 3 and Table 2. Two-way repeated measure ANCOVA showed that F–V and P–V potentials were both affected by angular acceleration (*P* < 0.001) and muscle structure (fascicle vs. muscle belly; *P* < 0.001). In particular, the fascicle and muscle belly F–V and P–V potentials decreased as a function of the angular acceleration, ranging from 0.12 ± 0.03 and 0.10 ± 0.03 to 0.07 ± 0.01 and 0.05 ± 0.01 for the muscle fascicle and muscle belly F–V potential, respectively; and from 0.70 ± 0.05 and 0.65 ± 0.06 to 0.56 ± 0.06 and 0.41 ± 0.07 for the muscle fascicle and muscle belly P–V potential, respectively. Finally, the F–V and P–V potentials of the fascicles are always higher than those reported by the muscle belly. However, for each explosive dynamic contraction, muscle belly reached faster shortening velocity compared to the fascicles.

### Determinants and Effect of Belly Gearing

**FIGURE 4:**
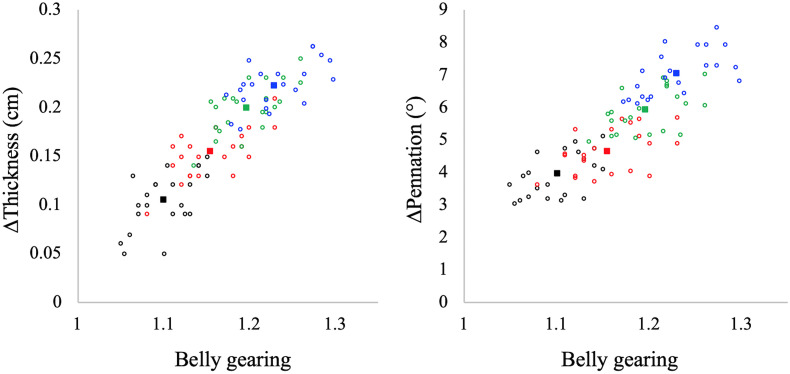
Muscle belly gearing as a function of muscle thickness (left panel) and pennation angle (right panel) changes in all the investigated contractions. Black: EDC_1000_, red: EDC_2000_, green: EDC_3000_, and blue: EDC_4000_. Open circles represent individual data points (*N* = 22 for each condition), while full squares represent the mean value for each condition. Hostelling’s test showed no significant differences between thickness and pennation angle in determining belly gearing.

**FIGURE 5:**
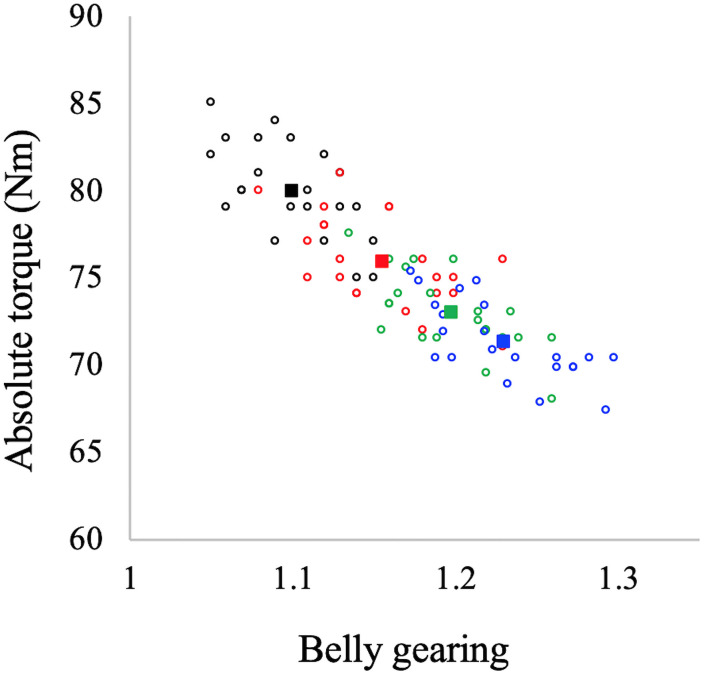
Belly gearing as a function of absolute torque. Black dots: EDC_1000_, red dots: EDC_2000_, green dots: EDC_3000_, and blue dots: EDC_4000_. Open circles represent individual data points (*N* = 22 for each condition), while the full squares represent the mean value for each condition.

In Figure 4 the values of belly gearing at any given explosive conditions are reported as a function of muscle thickness and pennation angle changes: the larger the changes in muscle thickness and pennation angle the larger the belly gearing. Hostelling’s test showed no significant differences between relationships (Gb-thickness vs. Gb-pennation). In Figure 5 the values of absolute torque are reported as a function of belly gearing. Belly gearing decreased as a function of the absolute torque.

## Discussion

This study aimed to determine the influence of belly gearing (and its determinants) on explosive torque during *in vivo* explosive concentric contraction.

In line with our hypotheses, the results revealed that the changes in muscle thickness play an important role in determining belly gearing during explosive dynamic contractions. However, we observed that the changes in belly gearing could not be explained by the changes in muscle thickness alone, but rather, by an interplay between thickness and pennation angle. As hypothesised, we found that belly gearing could allow to circumvent the limits imposed by the F–V and P–V relationships during low-force high-velocity contractions (from 1000 to 4000°/s^2^), allowing the muscle fascicles to operate at higher F–V and P–V potential compared to the muscle belly. In this regard, our results showed that when explosive contraction velocity increases (in the investigated range), belly gearing increases as well, increasing muscle belly velocity and allowing the fascicles to counteract the loss of torque. Without belly gearing, muscle fascicles would be forced to operate at lower potentials, with a decrease in the muscle’s mechanical output.

### Determinants and Effect of Belly Gearing

Muscles are isovolumetric and can bulge in thickness, width, or both, and the direction of shape change mediates the change in pennation angle during a contraction. During contraction, muscle fascicles rotate to a high pennation angle to create space for fascicles to be expanded (Herbert and Gandevia, 1995; Kawakami et al., 1998; Maganaris et al., 1998). Higher rotation enables muscle belly velocity to exceed that of muscle fascicles, allowing belly gearing to increase (Azizi et al., 2002, 2008; Brainerd and Azizi, 2005). Since there is some variation in how a muscle changes shape, and since this determines belly gearing, there is no single relationship between the length changes of a fascicle and that of the muscle belly (Wakeling et al., 2011; Eng et al., 2018). In accordance with the literature (e.g., Azizi et al., 2008; Wakeling et al., 2011; Holt et al., 2016; Dick and Wakeling, 2017), our data indicate that belly gearing is higher the lower the muscle force (see Figure 5). In particular, during explosive concentric contraction, belly shortening velocity is higher compared to that of fascicles in all the investigated conditions, resulting in a belly gear greater than 1. Moreover, our data indicate that belly gearing is positively correlated with the changes in pennation angle as well as with the changes in muscle thickness [as previously observed by Monte (2020)], suggesting that during explosive dynamic concentric contraction, belly gearing depends on the interplay between changes in thickness and changes in pennation angle. Finally, in agreement with the literature (Azizi et al., 2008; Holt et al., 2016; Monte, 2020), the changes in belly gearing are not related to changes in EMG activity (e.g., EMG amplitude was not affected by contraction speed, whereas this was not the case for belly gearing). Indeed, variable gearing results from the interaction between contractile and connective tissues properties, and is rather unaffected by neural parameters (e.g., Azizi et al., 2008; Holt et al., 2016; Monte, 2020).

In accordance with the F–V relationship, whereas the absolute value of torque decreased as a function of explosive contraction velocity, fascicles, and belly shortening velocity increase with it. It has been, however, suggested that belly gearing could allow to attenuate the limit imposed by the fascicles F–V relationship (e.g., Azizi et al., 2008; Wakeling et al., 2011; Eng et al., 2018; Roberts et al., 2019). Our data support this assumption. In fact, even if both fascicles and muscle belly F–V and P–V potentials decrease when the contraction speed increases, fascicles potentials are always greater than those of muscle belly. The positive effect of belly gearing could be observed in Figure 3: as far as the F–V relationship is concerned, it is possible to observe that, for the same muscle’s mechanical output (e.g., torque), muscle fascicles operate at a higher F–V potential compared to the muscle belly. Therefore, in all the investigated conditions, muscle belly velocity exceeds that of muscle fascicles, allowing the fascicles to operate at higher F–V potential. These results are in agreement with those reported by Azizi et al. (2008) during *in vitro* isotonic contractions. Concerning the P–V relationship (Figure 3): the fascicles P–V potential is higher than that of the muscle belly in all the investigated conditions. This result confirms the hypothesis that belly gearing could play an important role as “power amplifier” during muscle contraction (Brainerd and Azizi, 2005; Azizi et al., 2008), allowing muscle velocity to increase and the fascicles to maintain a high P–V potential. Therefore, even if nervous/neural factors have been suggested as the primary determinants of explosive force production (Del Vecchio et al., 2018, 2019; Tillin et al., 2018; Dideriksen et al., 2020), belly gearing could also play an important role, allowing to maximise the transfer between neural signal to joint motion.

### Methodological Consideration

This study presents some methodological issues. (i) We considered the VL muscle as the most representative of the knee extensors group (the quadriceps); however, the muscle force–length–velocity relationship could be different in the other knee extensor muscles. (ii) Our research set-up allows the muscle fascicles to operate at the same torque–length potential; however, even if the subjects started from rest, small changes in the starting position could affect the role of the elastic structures in the first milliseconds of contraction. These differences are expected to be negligible when the entire time window (150 ms) is considered, as done in this study. (iii) contractile history may cause force depression in pre-loaded concentric iso-velocity contractions, and this could influence the F–V curve and, therefore, the F–V potential during explosive contractions. This phenomenon (force depression) is proportional to the mechanical work done during shortening (Herzog and Leonard, 2000; Tillin et al., 2021) and is expected to be greater in a contraction with pre-activation than without pre-activation, as work will be greater in the former. In the current study we did not directly assessed the effect of force depression, but the torque values measured during the iso-velocity trials were only 1% higher than the estimated maximum isometric torque (T_*max*_). Furthermore, the values of torque collected during the explosive contractions never exceed T_*max*_. Finally, as recently reported by Tillin et al. (2021) explosive torque capacity seems to be unrelated with the effects of force depression. Therefore, our data of F–V and P–V potential during explosive contraction could be considered accurate.

## Conclusion

The present results provide novel evidence that belly gearing could affect explosive torque production during concentric contractions. Belly gearing allows the muscle to circumvent the limits imposed by the fascicle’s F–V and P–V relationships, increasing the shortening velocity and allowing the fascicles to operate at higher F–V and P–V potentials. This effect derives from the muscle’s possibility to change in shape. In particular, during explosive concentric contraction, the interplay between thickness and pennation angle (geometrical changes) allow belly gearing to increase as a function of contraction speed, thereby increasing the P–V potential and the torque rise, compared to a muscle without belly gearing (e.g., a fusiform muscle).

## Data Availability Statement

The raw data supporting the conclusions of this article will be made available by the authors, without undue reservation.

## Ethics Statement

The studies involving human participants were reviewed and approved by Ethical Committee of the University of Verona, protocol number: 2019-UNVRCLE-0193291. The patients/participants provided their written informed consent to participate in this study.

## Author Contributions

AM, MB, and PZ developed the idea and the experimental design. AM and RM collected the data. AM analysed the data. All authors contributed to both interpretation and discussion of the results and critically revised and edited the manuscript. All authors read and approved the submitted version.

## Conflict of Interest

The authors declare that the research was conducted in the absence of any commercial or financial relationships that could be construed as a potential conflict of interest.

## Publisher’s Note

All claims expressed in this article are solely those of the authors and do not necessarily represent those of their affiliated organizations, or those of the publisher, the editors and the reviewers. Any product that may be evaluated in this article, or claim that may be made by its manufacturer, is not guaranteed or endorsed by the publisher.

## Nomenclature

**Table:**

F–V	force–velocity
P–V	power–velocity
EDC	explosive dynamic contraction
RTD	rate of torque development
MVC	maximum voluntary contraction
VL	vastus lateralis
BI	biceps femoris
MVT	maximum voluntary torque
RMS	root-mean-square
T_*max*_	maximum torque
L_0_	optimal fascicle length
V_*f*_	fascicle velocity
V_*b*_	belly velocity
V_*MTU*_	muscle-tendon unit velocity
Gb	belly gearing
Fmax	maximum isometric voluntary force
Vfmax	maximal fascicle shortening velocity
Vbmax	maximum belly shortening velocity.
