# Supplementary material for: Muscle Belly Gearing Positively Affects the Force–Velocity and Power–Velocity Relationships During Explosive Dynamic Contractions
Source: Front Physiol. 2021 Aug 12;12:683931. doi: 10.3389/fphys.2021.683931 (PMC8387943; doi:10.3389/fphys.2021.683931)
Supplement: Supplementary file 1 [file Data_Sheet_1.pdf]

Muscle belly gearing positively affects the Force-Velocity and Power-Velocity relationships during explosive dynamic contractions

Andrea Monte, Matteo Bertucco, Riccardo Magris, Paola Zamparo

Supplementary Materials

RELIABILITY AND SENSITIVITY OF FASCICLE and MTU LENGTH MEASUREMENTS

We assessed the reliability and sensitivity of our fascicle length measurements by means of coefficient of multiple correlations (CMC) and average root mean square differences (RMSD), as suggested by several authors (e.g. Raiteri et al., 2016; Raiteri et al., 2019).

CMC was used to test the reliability of all variables during the explosive contractions in two different occasions (the first and the second experimental session) in all subjects. The level of significance was set at $\alpha = 0.05$. The within CMC value refers to the overall mean effect obtained in the two sessions, whereas the between CMC compared the overall effect obtained in the first experimental session and in the second session. RMSD values were calculated for both sessions to quantify the variability between trials.

The reliability analysis for all variables revealed high within-day and between-day CMCs (see Table S1). The RMSD values are reported in Table S2. No significant differences were observed between testing days for the investigated variables at all speeds.

Table S1*: Coefficient of multiple correlation (CMC) within and between tests for the investigated parameters. Data are means ± SD*

Fascicle length	
Within CMCs	*Between CMCs*
0.953±0.022	0.931±0.012

Table S2*: Average root mean square differences (RMSD) calculated to quantify the variability between trials. Data are means ± SD*

Fascicle length
Between RMSD
0.99±0.16

The systematic reliability and sensitivity assessment of the ultrasound-based measurements revealed excellent results for each of the analysed parameter. These results are in agreement with those obtained by other authors in different contraction types (e.g. Raiteri et al., 2016).

Figure S1. Ultrasound images during explosive concentric contraction as visualised using the semi-automatic software (UltraTrack).

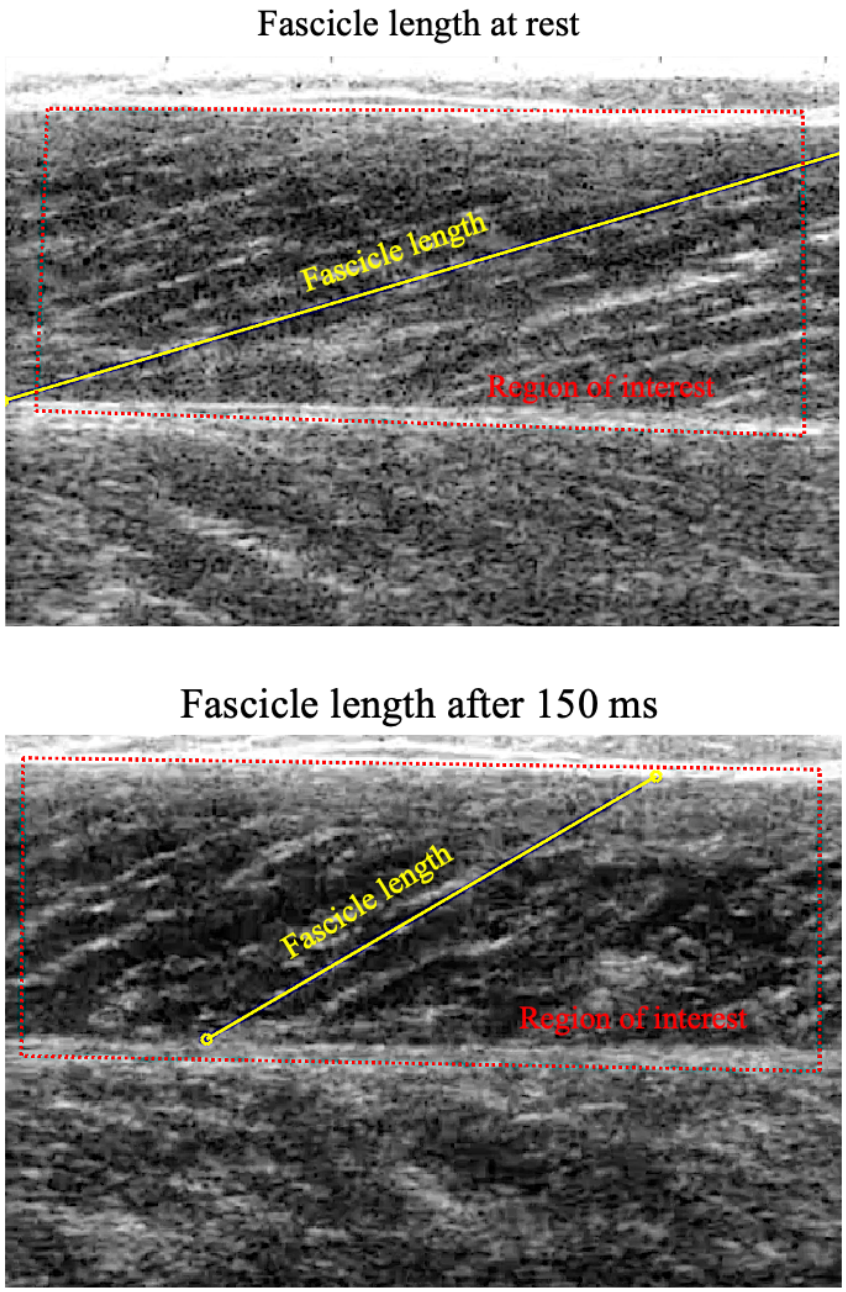

Figure S1: ultrasound images during rest and after 150 ms from torque onset during an explosive dynamic contraction at 3000°/s^2. Fascicle length is reported in yellow and the region of interest (ROI) is highlight in red.

Table S3. Individual values obtained from the T-L, T-angle and T-V relationships.

	T_{max} (N m)	L_0 (cm)	Optimal angle (°)	Vf_{max} (cm/s)	Vb_{max} (cm/s)
S1	310	7.9	63	55.4	73.4
S2	254	9.1	65	42.4	58.4
S3	301	10.11	67	49.1	61.1
S4	290	8.44	68	58.3	70.3
S5	277	7.3	61	53.6	69.6
S6	302	9.09	69	45.9	33.9
S7	305	9.66	67	54.7	66.7
S8	266	10.99	65	44.5	54.5
S9	240	11.03	65	49.9	68.9
S10	245	11.11	63	56.2	76.2
S11	253	7.55	67	47.9	65.9
S12	265	8.01	68	43.6	54.6
S13	299	7.77	68	48.2	62.2
S14	283	8.88	66	49.3	63.3
S15	274	8.21	65	57.9	70.9
S16	300	11.35	63	62.0	71.5
S17	261	9.11	62	60.1	77.1
S18	264	9.46	66	41.3	59.3
S19	285	9.56	68	43.8	55.8
S20	311	9.5	65	55.7	71.7
S21	250	10.11	65	49.6	65.6
S22	246	10.77	67	51.2	68.2

Footnote: T_{max}: maximum isometric torque obtained from the T-L relationship; L_0: optimal fascicle length obtained from the T-L relationship; Vf_{max} and Vb_{max}: maximum fascicle and belly shortening velocity obtained from the T-V relationships.
